# From ivory tower to inclusion: Stakeholders’ experiences of community engagement in Australian autism research

**DOI:** 10.3389/fpsyg.2022.876990

**Published:** 2022-08-25

**Authors:** Jacquiline den Houting, Julianne Higgins, Kathy Isaacs, Joanne Mahony, Elizabeth Pellicano

**Affiliations:** ^1^Macquarie School of Education, Macquarie University, Sydney, NSW, Australia; ^2^Cooperative Research Centre for Living With Autism (Autism CRC), Brisbane, QLD, Australia; ^3^Sylvia Rodger Academy, Cooperative Research Centre for Living With Autism (Autism CRC), Brisbane, QLD, Australia; ^4^Department of Developmental Disability Neuropsychiatry (3DN), University of New South Wales, Sydney, NSW, Australia; ^5^The Autistic Realm Australia, Inc., Adelaide, SA, Australia; ^6^Department of Clinical, Educational and Health Psychology, University College London, London, United Kingdom

**Keywords:** patient and public involvement, autism, community engagement, participatory research, co-production

## Abstract

Autistic people, and other community stakeholders, are gaining increasing recognition as valuable contributors to autism research, resulting in a growing corpus of participatory autism research. Yet, we know little about the ways in which stakeholders practice and experience community engagement in autism research. In this study, we interviewed 20 stakeholders (academics, autistic people, family members/careers, research students, and service providers) regarding their experiences of community engagement in Australian autism research. Through reflexive thematic analysis of interview data, we generated four themes. First, our participants perceived academia as an “ivory tower,” disconnected from community members’ lives and priorities. Second, our participants identified that different stakeholders tended to hold different roles within their research projects: academics typically retained power and control, while community members’ roles tended toward tokenism. Third, our participants spoke of the need to “bridge the gap” between academia and the community, highlighting communication, accessibility, and planning as key to conducting effective participatory research. Lastly, participants emphasized the changing nature of autism research, describing participatory research as “the way of the future.” Our findings reflect both the progress achieved to date, and the challenges that lie ahead, as the field advances toward genuine co-production of autism research.

## Introduction

Over the past decade, there has been increasing recognition of the value of community engagement in autism research. As recently as 2014, examples of participatory research were rare in the autism literature (Jivraj et al., 2014). Following trends in health research and a range of other fields, however, a growing body of participatory autism research has developed since that time (see Crane et al., 2019; Fletcher-Watson et al., 2019; Nicolaidis et al., 2019; Benevides et al., 2020 and Keating, 2021, for further discussion and examples).

The term *participatory research* refers to research conducted with meaningful input from members of the relevant community/ies during the research process. In autism research, this typically involves academics (research professionals and/or research students) working together with community members (autistic people; their families, friends, and carers; service providers; and other stakeholders) to produce research. Ideally, community members will be involved across all stages of the research process, sharing power and control as equal partners in a research team—a participatory approach known as *co-production* (Filipe et al., 2017; Roper et al., 2018; Redman et al., 2021).

In Australia, the Cooperative Research Centre for Living with Autism (Autism CRC) has contributed a considerable proportion (approximately 45%) of national autism research funding since its establishment in 2013 (den Houting and Pellicano, 2019). Autism CRC is the world’s first national, cooperative research effort focused on autism, and comprises a collaborative network of more than 50 participant and partner organizations, including universities, autism service providers, autistic and other advocacy organizations, industry entities, and government departments (Autism CRC, 2021). Autism CRC promotes inclusive and community-engaged research practices, with a strong focus on research co-production. Autism CRC has established a range of initiatives to promote and incentivize participatory research including, for example, the Participatory and Inclusive Autism Research Practice Guides (den Houting, 2021), which provide information and guidance regarding the conduct of participatory autism research; the Co-Production Partner Initiative, which recognizes organizations that show a commitment to sustainable research co-production (Autism CRC, 2022a); and the Sylvia Rodger Academy Research Program, which provides training to equip researchers and autistic adults with the skills needed to co-produce autism research (Autism CRC, 2022b). In previous research, we examined the extent and nature of community engagement in research commissioned by Autism CRC (den Houting et al., 2021), using an online survey. We identified that, while Autism CRC stakeholders expressed strong support for community engagement in research, these positive attitudes often failed to translate into participatory research practices. Our findings suggested that there remain barriers— in particular, systemic constraints and knowledge gaps regarding participatory research—limiting the conduct of high-quality participatory autism research in Australia.

While community engagement in autism research continues to increase, our understanding of the attitudes and beliefs informing such engagement remains limited. To our knowledge, the first investigation of attitudes toward community engagement in autism research was conducted by Pellicano et al. (2014), who gathered both researcher and community views. Overall, researchers reported engaging with the autism community to a moderate extent, while community members reported significantly lower levels of engagement. Researchers held varied opinions regarding the value of community engagement, with some believing that community input should be central to the research process, while others felt that research should remain in the hands of scientists. Researchers were concerned that there is a lack of diversity among the community members who are most frequently engaged in research, and felt that autistic characteristics can make it difficult to work with autistic people. Community members, in contrast, felt that their contributions to research were often undervalued. Some described a lack of opportunities for engagement; others described one-sided engagement during which they were “treated like guinea pigs” (Pellicano et al., 2014, p. 7). As a result, community members found that research findings were often inaccessible, and lacked relevance to their daily lives.

More recently, both Hollin and Pearce (2019) and Pickard et al. (2022) elicited autism researchers’ attitudes toward community engagement in research. In both studies, participants reported the belief that community insights are valuable, but also voiced a range of concerns. Hollin and Pearce’s participants described challenges they encountered when working with autistic people and tended to attribute these challenges to autistic characteristics; for example, stating that disagreements between autistic and academic stakeholders arose due to autistic people’s perceived impairments in perspective-taking. Pickard et al.’s participants noted similar communication challenges, which they attributed as resulting both from autistic characteristics and community members’ unfamiliarity with research. Participants in both studies were concerned that the autistic people who contribute most frequently to participatory research may not fully represent the diversity of the autistic community. At the same time, though, participants expressed confusion regarding how to respond to the at-times conflicting views held by different autistic stakeholders. Additionally, while Pickard et al.’s participants believed that participatory approaches are becoming more common in autism research, they noted that this shift toward increased community engagement may be hindered by considerable systemic barriers and a confusing lack of clarity surrounding participatory research terminology and practices.

To examine experiences of participatory research from the perspective of research participants, Pellicano et al. (2022) interviewed autistic adults who had taken part in the Hidden Histories project, an oral history research project co-produced by a team of autistic and non-autistic researchers. Almost universally, participants in the Hidden Histories project felt that the involvement of autistic researchers had improved their experience as participants. Indeed, for some participants, the co-produced nature of the project was a key factor in their decision to take part, as it provided reassurance that the research ethos was aligned with participants’ own values and priorities. Participants described feeling supported by the research team throughout the study, and were able to form connections with the autistic researchers (who conducted the oral history interviews) that would be less likely to develop with a non-autistic researcher. As a result, participants felt safe and comfortable sharing their stories, despite the often-confronting content of their narratives.

These studies have provided preliminary insights into stakeholders’ perceptions and experiences of participatory autism research. With the growing trend toward participatory research in this field, though, deeper understanding will be vital in informing future community engagement. In this study, we focused on the practicalities of participants’ involvement in participatory research, to elucidate the factors they perceive to have most shaped these experiences. We examined (1) how stakeholders practiced and experienced participatory research within Autism CRC research projects; (2) why participatory research was practiced and experienced in this way; and (3) how we might improve participatory autism research going forward.

## Materials and methods

### Participants

Participants in this study comprised a sub-sample of participants from a previous online survey study examining perceptions of participatory autism research in Australia (den Houting et al., 2021). Recruitment was initiated by Autism CRC, who contacted all Autism CRC Project Leaders with a request to nominate current and previous members of their research team/s (including both academic and community stakeholders) for participation in the online survey. Project Leaders and nominated team members were invited to complete an online survey, with the option to participate in a follow-up interview. Of the 79 participants who completed the survey, 25 consented to being contacted for participation in a follow-up interview. Of those 25, 20 participants (80%) took part in the interview (four did not respond to email invitations and one declined to participate).

Each participant completed a brief demographic questionnaire prior to interview (see Table 1). Sixteen participants (80%) were women, three (15%) were men, and one reported non-binary gender. Participants’ ages ranged from 24 to 72 years (*M* = 45.15, SD = 13.69). All reported some tertiary education, with more than half (55%) holding a PhD or Doctoral degree, and most (70%) engaged in full-time employment or study. Most participants (70%) reported a white European racial background. Participants identified their various (often multiple) roles within the autism community, with 15 identifying as an autism researcher; six as a family member or carer of an autistic person; four as an autistic person; four as service providers; and three as research students studying autism.

**Table 1:** Participants’ demographic characteristics.

Participant ID	Gender	Age	Education	Occupation	Role/s in autism community
01-R	Woman	33	PhD/Doctorate	Full-time employment	Researcher studying autism
02-A	Man	69	VET*, Diploma, or Associate Degree	Retired	Autistic person
03-StF	Woman	40	Master’s Degree or Postgraduate Diploma	Full-time study	Student studying autism; family member/carer of autistic person
04-R	Woman	54	PhD/Doctorate	Full-time employment	Researcher studying autism
05-StF	Woman	24	Bachelor’s Degree	Full-time study	Student studying autism; family member/carer of autistic person
06-RSp	Woman	36	PhD/Doctorate	Full-time employment	Researcher studying autism; service provider
07-AR	Woman	35	Master’s Degree or Postgraduate Diploma	Part-time employment	Autistic person; researcher studying autism
08-R	Woman	35	PhD/Doctorate	Full-time employment	Researcher studying autism
09-RSp	Woman	59	PhD/Doctorate	Full-time employment	Researcher studying autism; service provider
10-F	Woman	49	Bachelor’s Degree	Full-time carer/domestic duties	Family member/carer of autistic person
11-R	Man	54	PhD/Doctorate	Full-time employment	Researcher studying autism
12-R	Man	38	PhD/Doctorate	Full-time employment	Researcher studying autism
13-ARSp	Woman	31	Bachelor’s Degree	Full-time employment	Autistic person; researcher studying autism; service provider
14-RF	Woman	46	PhD/Doctorate	Part-time/casual employment	Researcher studying autism; family member/carer of autistic person
15-AR	Non-binary	72	Bachelor’s Degree	Part-time/casual employment	Autistic person; researcher studying autism
16-StSp	Woman	28	Bachelor’s Degree	Full-time study & part-time employment	Student studying autism; service provider
17-RF	Woman	40	Bachelor’s Degree	Part-time employment & part-time study	Researcher studying autism; family member/carer of autistic person
18-RSp	Woman	63	PhD/Doctorate	Full-time employment	Researcher studying autism; service provider
19-RF	Woman	55	PhD/Doctorate	Full-time employment	Researcher studying autism; family member/carer of autistic person
20-R	Woman	42	PhD/Doctorate	Full-time employment	Researcher studying autism

* Vocational education and training.

### Interviews

Each participant took part in one semi-structured interview, either via Zoom (n = 17) or face-to-face (n = 3). All interviews were conducted by the first author, an autistic early-career academic. Interviews ranged from 38 to 77 min in length (M = 54 min). Nineteen interviews were audio recorded, using Zoom’s inbuilt recording function and/or a digital voice recorder, and transcribed by a professional transcription service. One participant did not consent to being recorded, and the interviewer took notes by hand during this interview. All transcripts were returned to participants for review and correction prior to analysis. Community stakeholders were offered a AUD$20 gift card for their participation (participants employed in paid roles in autism research did not receive gift cards).

During the semi-structured interview, we asked participants to describe their own personal and professional experience with autism and, specifically, autism research. We asked them to describe their understanding of participatory research, and how participatory research differs from more traditional research. Next, we asked participants to bring to mind their experience of one specific Autism CRC research project of their choosing, and to describe this research process. We asked them to explain whether and how community members were involved in their research process, to describe the relationships between different stakeholders in their research process, and to describe the outcomes of their research process. We also asked about the benefits and challenges of community engagement within their specific research project, and how community engagement impacted their research project. Lastly, we asked participants to talk about autism research more generally. We asked questions about historical and current perceptions of autism research, perceptions of autistic people’s roles in research, and ways to improve meaningful community engagement in autism research (see Supplementary material).

### Data analysis

Data were analyzed using reflexive thematic analysis. We approached analysis through an interpretivist/constructivist perspective, recognizing that individuals create meaning, with each person’s individual reality influenced by their social context. In so doing, we approached analysis with the understanding that, just as our data reflected participants’ contextually situated experiences, our analyses reflect our own contextually bound interpretations of the data. The community of autism researchers in Australia is relatively small and well-connected, meaning that many of our participants were acquainted with the interviewer prior to taking part in this study. Our findings should be considered within this immediate interview context, as well as broader academic and social contexts. We adopted an experiential orientation, with language (and therefore our data) assumed to accurately reflect participants’ constructions of their experiences and realities (Braun and Clarke, 2021).

The first author is an autistic early-career researcher and activist, with expertise and experience in participatory autism research. This author led analysis, working through Braun and Clarke’s six-phase reflexive approach: (1) familiarization with the data; (2) generating initial codes; (3) searching for themes; (4) reviewing potential themes; (5) defining and naming themes; and (6) producing the manuscript (Braun and Clarke, 2012, 2019; Braun et al., 2019). Because many of our participants identified with multiple roles in the autism community, it was not possible to create distinct participant groups (e.g., academics versus community members); therefore, data from all participants were analyzed collectively.

After familiarizing themselves with the data, the first author generated and applied codes to each transcript using NVivo version 12. In line with our experiential orientation, we coded the data at the semantic level, based on the explicit meanings of the data. We coded data inductively, aiming to construct codes from the data, rather than from pre-existing knowledge or theories. After coding all transcripts, the first author developed preliminary themes by collating similar codes and discarding codes that did not appear relevant to the research questions. This process produced 12 candidate themes, which were explored through thematic mapping. Through re-engaging with the data coded within each candidate theme, the preliminary themes were revised and a thematic structure consisting of five themes and nine subthemes was constructed. Next, the first author selected data extracts to illustrate each subtheme, and sought participants’ approval to publish the relevant de-identified extracts from their interview transcripts in this manuscript. The first author then generated descriptive names for each theme and subtheme.

**Figure 1:**
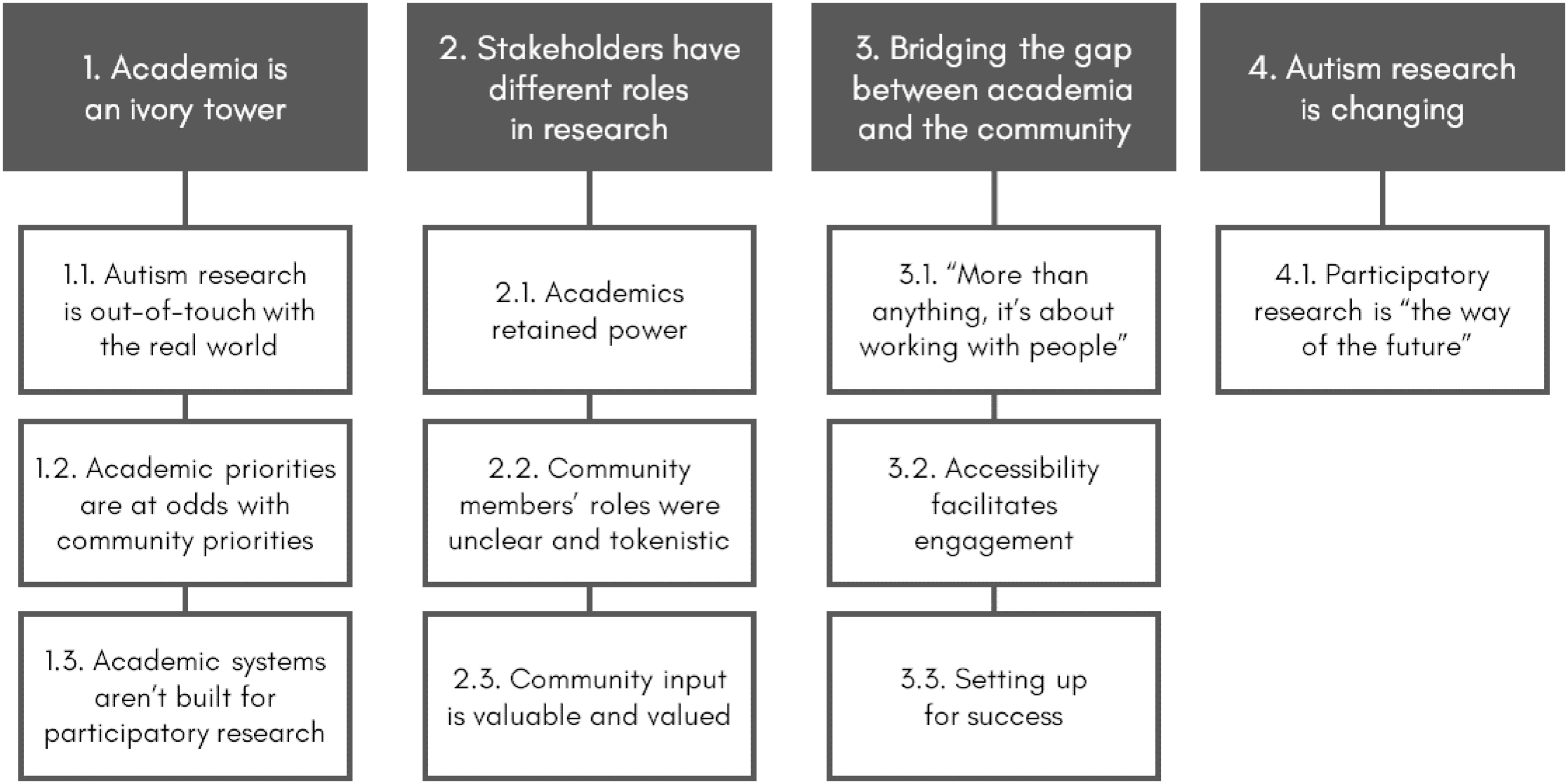
Participatory autism research practices and experiences: themes and subthemes.

With input from the last author, an experienced non-autistic researcher with expertise and experience in participatory autism research, the first author produced a draft of this manuscript. During the writing process, it became clear that one of the five themes was better conceptualized as a subtheme within one of the other themes. The themes were revised accordingly, resulting in the current thematic structure of four themes and ten subthemes (Figure 1).

### Community engagement in this study

Members of the autistic community were engaged throughout the process of this research project. The project plan (including funding application) for this study was developed by the last author, in consultation with the first author. An Autistic Advisory Group, consisting of five autistic adults with experience as both peer researchers and research participants, was established during the early stages of this project. The Advisory Group acted as consultants throughout this study, providing input via web conferencing and email.

The semi-structured interview template was initially developed by the first author, with input from the last author. This template was then revised based on feedback from the Advisory Group. The first author led participant recruitment, conducted all interviews, and led data analysis. Members of the Advisory Group contributed to this manuscript and are credited as authors, where appropriate. Advisory Group members were paid for the time they spent consulting on this project.

## Results

We developed four themes that addressed our research questions, described below. Quotes from individual participants are identified by participant number and the participant’s role/s in the autism community (R = researcher; A = autistic person; F = family member/carer; Sp = service provider; St = student).

### Theme 1: Academia is an ivory tower

#### Autism research is out-of-touch with the “real” world

Participants, including researchers, described the field of autism research as being largely out-of-touch with the realities of autistic people’s lives. They spoke of autism research as failing to address the autistic community’s priorities, instead being “more focused on things like genetics, or parent stress, that are quite stigmatising or… not vital to their day-to-day functioning” (06-RSp), or “addressing stuff that just does not matter. It’s just irrelevant. It just does not matter” (04-R). Participants felt that autism research often failed to improve the lives of autistic participants, who were “just… contributing their information, contributing their experiences to studies that… would never help them, in the end” (05-StF).

Academics themselves were described as similarly out-of-touch with autistic people’s experiences: “I mistakenly thought that researchers would know a lot about autism, but they know a lot about autism research and that does not mean to say they know a lot about autism, the lived experience” (15-AR). Beyond autism itself, academics were perceived as disconnected from the community more generally. As one autistic person noted, “they are very much steeped in academia” (02-A). This participant described how,

You learn nothing more because all the conversation revolves around is the academia… Now, I can understand living in that sort of research type environment, it’s a bit like being an archeologist and wanting to be going away and scraping away in a trench all day long, oblivious of time and everything, because you can immerse yourself in it… They’re living their life through academia. (02-A)

Some researchers shared this sentiment:

It’s easy to get lost in the data, the ethics applications, the measures, the validity and the reliability, but actually constantly being reminded that these are people with real lives… If that’s not constantly reminded to you, it’s easy to get lost in that rabbit hole of academia. (01-R)

For one participant working in a basic science setting, it was important to introduce a sense of humanity into his team’s work:

There were people that… would deal with the samples and do the biological research, that would have no interaction whatsoever with that family. I try to instil into some of the research staff and students that work on these projects, that when you’re talking about the cells, you’re talking about… these are Peter’s cells, Peter is eight years old, he loves cars. You try and add that personal touch to it. This is actually a person that we’re working with. (11-R)

Some participants also noted the downfalls that can arise when an awareness of the real-world context is lacking in research. One participant, for example, perceived this to limit the utility of research outputs in community settings:

A lot of things developed in universities, they’ve used a lot of exclusion criteria and they’ve excluded all the people with challenges… they have communication difficulties or learning difficulties or whatever… So, we often end up having to adapt what universities have produced because it’s not meeting the needs of the people that come to us. Or we have to develop our own anyway. (18-RSp)

#### Academic priorities are at odds with community priorities

Participants often faced difficulties conducting research that met community needs, while also meeting academic expectations regarding scientific rigor: “There were certain rigidities around methodology, and certain rigidities in the classroom around curriculum and what has to be done, so there was a bit of negotiation having to happen to get those two things to work together” (14-RF). One participant was frustrated that rigid ideas around research methodology overshadowed the value of her work:

I keep getting feedback that, ‘Well, you didn’t do a randomized controlled trial’. Well, that’s not what this is about. This is about people being able to express what’s important to them, so how can I possibly do a randomized controlled trial? It’s really about individuals and what’s meaningful to them, so I can’t compare one person to another. There’s nothing standardized about it. (18-RSp)

Another participant described a similar experience:

With the focus being on something real-world and actually useful, I think the challenge as an academic is that it’s probably going to result in one paper that we’re probably going to have a hard time publishing because it’s real-world data, it’s messy, there’s missing data, sample sizes maybe aren’t perfect, it’s not a randomized controlled trial but… We’ve learnt a lot, and we’ve really tried to make it very real-world, and it’s been [difficult] trying to balance that with the demands of, you know, the whole ‘publish or perish’ and so on. (06-RSp)

Disparities between university priorities and the priorities of community organizations were highlighted as a particular challenge, with universities described as “all about the thesis and the student and… not terribly good at working with community organizations and thinking that you have anything to offer” (18-RSp), and community organizations perceived as being “unaware… of the processes and the checks that we had to go through for the PhD student at the university, those requirements from the university” (04-R). As one participant explained,

There were also competing priorities within the organization at the other end, because at a university level, you are here to do that research project, or you are teaching your research and that’s it. Whereas if you are a not-for-profit organization and you are running multiple research projects, plus all of the other things that you do, there’s a lot more priorities going on at one time. (16-StSp).

On an individual level, researchers felt that community members had “a very different sense of what research can be” (20-R), which led to difficulties in “getting the stakeholders to really understand what rules we, they, need to abide by” (04-R). Others, though, felt that it was academics’ responsibility to ensure scientific rigor in participatory research projects:

Sometimes you might need to be trying to explain to one of the advisors why… advice might not be able to work because of the context of the research methodology and things like this. I think that’s part of working together as a team. That’s what the researchers will bring – is that deeper understanding of methodologies and approaches to the science. That’s one of the skills of the researcher groups. (12-R)

#### Academic systems are not built for participatory research

Participants consistently reported that academic systems pose major barriers to the conduct of participatory research. One researcher spoke about academic processes as being “in opposition… to participatory research,” describing how they have to “figure out how to make [participatory research] fit within the existing protocols and systems” (04-R). Another participant agreed, stating,

I think organizational structures don’t reward that, too. Like if you’re thinking about what universities want on the time and space they provide, it’s really hard… They have structures around what people can and can’t do. So it’s often harder to engage in collaboration where you’ve got organizational rules about what can and can’t happen. (16-StSp)

Participants highlighted funding and time constraints: “I guess it’s still limited by issues like funding, just time constraints. The pressure… for people to just constantly publish to justify funding… those constraints make it difficult” (05-StF). More specifically, participants were concerned that the academic funding system lacks adequate provisions to ensure that community members—particularly autistic people—are paid appropriately for their work “right at the stage of writing the grant” (12-R). As one researcher noted,

If an autistic person is going to come in and contribute, they absolutely deserve to be paid for their time… it's part of the challenge… how to work that within an institution and a funding model and a grant and all of that sort of thing. (17-RF)

### Theme 2: Stakeholders have different roles in research

#### Academics retained power

Perhaps unsurprisingly, participants reported that researchers typically retained the majority of power within their research projects. As one participant described, “the decisions sat with the academic research team, really. Even though that should not have been the case, that’s how it worked… that power imbalance is definitely there, at the end of the day, if everyone’s honest” (01-R). One community member clearly described this power imbalance:

I admire all the credentials of those people and that, but it’s a question of whether you feel like you can contribute to that group, because it seems like there’s two levels. You know, you’ve got the researchers who are dominant, and you’ve got perhaps a few others that have had a lot of life experiences and are certainly far from unintelligent… but are unable to meet on the same plane. (02-A)

Often, power dynamics followed a hierarchical structure, with Chief Investigators holding the balance of power, “I think the head of the project definitely had the majority of the power. Most people were kind of just looking to her to say, okay, well, what do we need to do next?” (08-R). Some participants felt that this was necessary to ensure projects ran efficiently, “Of course, at the end of the day the leader—the research team needs to make… those decisions” (20-R), noting that such decisions would involve a “negotiation process” (20-R) with community members.

Some participants were aware that the existing power dynamics are not in line with best practices for participatory research, “the power exchanges, because it’s collaborative and it’s genuinely – well, it should be, I’ll clarify that – should be a genuine power sharing. So, there should not be, ‘well, the academics hold more power because they came up with the initiative’” (16-StSp). Despite acknowledging that “you have to let go of control” (04-R), however, some researchers held concerns about the practicalities of sharing power:

Letting go of control is one thing, but how much control do you need to let go of and how much do you still need to maintain? Because sometimes when you get too participatory, nothing ever happens. There's too many voices, they never come to a decision. (04-R)

Importantly, participants expressed the belief that, within autism research, there are some “academics who are incredibly resistant and do not want to change things at all… who do not want to let go of their power. They like things done a certain way, and they like the way things work right now and the current reward structures” (06-RSp).

#### Community members’ roles were unclear and tokenistic

Some participants expressed a lack of clarity regarding community members’ intended roles within their various research projects. Community members, in particular, were uncertain of how they were expected to contribute to research: “What are we supposed to be doing? Are we advisors or are we actually providing the material [data] that you can use?” (02-A). They were even unclear about the nature of the research that they were involved in:

I did one [advisory role] and then I think one of those may have merged into something else, I think. So, I think it was two [projects]… I think it was somebody's PhD. I think it was her – I’m almost certain it was her PhD research. (10-F)

Community members typically held advisory or ancillary roles and were perceived by researchers as “external collaborators rather than project team members” (14-RF). This perception was consistent with community members’ own experiences, “I would not say that I felt a part of a team. Definitely not, and I would have liked to” (10-F). Community members described working in isolation from other project stakeholders, “I do not know anyone who was involved in it… I only know them by their name, but they could just—they could walk past me now, I would not know” (10-F), often performing their roles “via the computer… through emails and transfer of documents” (02-A). Some participants felt that community members were not given “the opportunity… to really influence or change how the project worked in any way” (16-StSp), and community members agreed that they “definitely would have liked… probably a stronger input or influence” (10-F). The lack of clarity regarding one participant’s role, and lack of engagement with the wider project team, left him “feeling sometimes that it is almost like I am the token autistic person that is not involved, like all the others, in research professionally” (02-A).

Consistent with the ancillary roles that community members often played, some participants questioned whether attempts at community engagement were genuine:

I feel like each time they say ‘we need to ask the autistic community’, it’s… ‘oh, we have to do this, or else we might get in trouble’. It feels like… we’re only doing it because we’re being told we have to do it. (03-StF)

Another participant felt that community members were engaged “when it suited the organization’s aims to look participatory,” noting that there wasn’t “a genuine acknowledgement across the board that these people brought expertise to the project, that could have been used in a lot of different ways” (16-StSp). An autistic researcher described this *ad hoc* consultation as a common experience, “‘Oh, can you just read the survey and make sure that autistic people are not going to get upset about my language?’ It’s like, ‘Yeah, I can do a lot more than that’” (13-ARSp).

#### Community input is valuable and valued

In contrast, many researchers reported that they highly valued community members’ input. Participants felt that community input assisted them in “understanding the needs of individuals” (11-R), and “brings in a whole new perspective [that has] been extraordinarily valuable to what we do. It makes the team think about how we go about doing research, and about our priorities” (09-RSp). Some autistic participants shared these positive perceptions, feeling that their “input was valued and that it was a genuine part in shaping the research” (07-AR).

In particular, participants described community engagement as having beneficial impacts on research findings, outputs, and outcomes. One participant stated, “I wished that they were brought in earlier, to help me with the process, just because I thought they were so valuable to my interpretations in the end” (05-StF), while others felt that input from community members had “really made a big impact on the findings” (12-R), “helped to ensure that the study that I’m doing is worth it” (03-StF), and demonstrated “how much better outcomes and results you can get… when you do get involved with a bigger range of stakeholders” (17-RF).

In some cases, community members were engaged due to having a particular skillset relevant to a research project, and therefore “contributed to the areas that they were most passionate about and… actually drove a lot of those areas… that was a great benefit to have that in the project” (06-RSp). In other cases, community members’ insights into the lived experience of autism were an asset that academics prized:

I think there’s perception, potentially, from them that they have to have some sort of skillset related [to research]; they have to be able to read academically or write academically… it's like, maybe some people don’t actually know the value that their experience of day to day, that’s actually invaluable to us and that’s exactly what we’re trying to get at. It’s not about whether you can read an academic paper or not or whether you might understand the statistical approach. (01-R)

Despite these generally positive attitudes toward participatory autism research, some participants felt that there remains “room for improvement” (18-RSp), with “steps to be made in making sure participation is even more valued and has even more, kind of, concrete contributions” (17-RF). One participant noted that this is not unique to autism research, but extends across the broader disability community: “[we need] almost an attitudinal shift about the value of people with disabilities’ voices… we are not very good at that, if you look across any of the disability groupings, we do not value [their voices]” (16-StSp).

### Theme 3: Bridging the gap between academia and the community

#### “More than anything, it’s about working with people”

For some participants, the diversity of academic and community perspectives was managed by simply working together as people. Participants described the processes of communication and relationship-building that took place between different stakeholders in their research projects, noting that “really open communication was key” (17-RF), and that “there’s no shortcut to building trust in good relationships” (07-AR). For some participants, developing a positive working relationship with other stakeholders was relatively easy, “We’ve had really good rapport I think… [I] got to know the team there, I think, pretty well and had no hesitation… I think we built up a pretty good relationship” (06-RSp). Some participants connected over shared experiences, “I feel real empathy for her and with what maybe she is going through… we were in tune together, which was nice” (02-A). Others were able to work together to build strong and meaningful partnerships over time:

I think that we actually work well as a team… we’re able to be upfront with each other and also we were able to support each other through it… We’ve got the opportunity to learn from each other, you know, and we do appreciate each other’s strengths. (15-AR)

One research student described benefitting from her team’s established relationships with community members, which facilitated open communication and collaboration:

It was good that there was already an established network of advisors… there’s already a relationship between the [research] team and them, so the communication felt open, almost in some cases very friendly and conversational… they weren’t afraid of making suggestions, because they already knew the team would be open to them… it was a strong relationship between the team and the advisors, and that paved the way for it to be more of a collaborative thing. (05-StF).

For many participants, though, communication between stakeholders was a source of tension. Occasionally there were difficulties with communication across neurotypes, “there are some challenges with communication obviously on the autistic side, but also on that neurotypical side of things, people—they have their own communication quirks and it does not always work” (13-ARSp). One autistic participant described his academic peers’ communication as overly “formal… rigid… just, bloody get on with it” (02-A). These challenges were not limited to communication between autistic and non-autistic stakeholders, however, with participants equally often describing tensions arising between non-autistic team members from different academic and/or community organizations. Participants felt that these conflicts reflected “a problem of perspective” (04-R) due to different professionals “not understanding each other’s ways” (09-RSp):

I think that [relationships were] an ongoing challenge and struggle as well, because I think there was definitely a perception of very differing aims from different departments… then add on top of that, different personalities, just of the human kind, which really did influence the politics of working through some of these projects. (01-R)

#### Accessibility facilitates engagement

Accessibility was frequently highlighted as a priority for research involving autistic people as team members or consultants. One autistic participant noted that the process of community engagement should be approached with as much care as the research process itself: “The projects themselves are very important, but there should be equal amount of thought put into the actual community meetings or the input of individuals… the mechanics if you like, need to be really looked at” (02-A). Another participant spoke of her annoyance upon witnessing meetings conducted without necessary accommodations for autistic team members:

I think it’s just good meeting practice to make sure it’s inclusive of who’s in a space… some of those things didn’t happen, which then actively excluded or made it difficult for the people on the spectrum to participate… I found it frustrating to be in a room where we weren’t setting things up for people to actually be part of the process. (16-StSp)

Encouragingly, many researchers spoke about the strategies in place within their projects to facilitate accessibility for community members, and autistic people in particular. Participants described a wide range of strategies, including accommodations for sensory processing differences (“Somebody says it’s too bright or it’s too noisy, there were adjustments. ‘Can I wear ear plugs?’ ‘Yes, of course you can wear ear plugs, can we buy them for you?’ So we get some noise cancelling ear plugs”; 09-RSp); preferences regarding social interaction (“One of our advisors – maybe more than one – has actually commented that they actually prefer not having a group interaction situation… They’re quite happy to provide their feedback individually to researchers working on the project”; 12-R); executive functioning difficulties (“doing things like sending a reminder the day before”; RSp-06); and differing levels of education and cognitive ability (“We put together a template of how you might present the results [to community members] … You’d still include potentially the table of numbers, but then under it would be a blurb written in not necessarily strict academic writing”; 01-R). Regarding which specific strategies to implement, participants noted the importance of being “flexible, and understanding that every autistic person… will have different preferences for the way that they engage with the project… being aware of that and changing your approach” (12-R).

#### Setting up for success

Participants explained that, to conduct effective participatory research, community engagement needs to occur “right from the beginning” (04-R) of a research project. Processes for community engagement must be deliberately planned as a core component of the research,

I think you have to build it in from the get-go. You can’t retro fit it. You can’t add it on as something that looks good or meets a requirement because neither of those are going to be genuine and they’re not going to work… You need to plan in the power structures and the power sharing, so that that’s actually intentionally done, rather than just kind of ad hoc approaching things. (16-StSp)

This planning process should involve all relevant stakeholder groups, and take into account the research context and the individual needs of each stakeholder, “establishing early on how, when, why the engagement is going to happen and how people want the engagement to happen, both the autistic individuals and the researchers… it’s got to be individual to every project and every person” (12-R).

Participants acknowledged that conducting high-quality participatory research can require considerable effort but felt that investment was justified by the potential benefits: “when the academics are willing, you can really see the effort they put in… it becomes a smoother process. Just that willingness to bridge that gap makes a huge difference” (05-StF). Some found benefit in departing from typical research processes to engage creatively with different stakeholders: “What this project has shown is, if you do think creatively about ways that people can express themselves in different ways… then they can be a lot more involved, actively involved” (18-RSp). One participant explained the importance of planning and sustained effort for facilitating inclusion:

If the structures are right, anybody can participate at any time, but it has to be set up for people to be successful… You take little steps, and you keep trying and you keep doing more, and you keep building skills and capacity, and then eventually, you’ll get there. (16-StSp)

### Theme 4: Autism research is changing

Participants overwhelmingly agreed that “autism research is really in a changing space” (16-StSp), describing “a huge amount of change in our knowledge in the field, and how we look at autistic people” (04-R). Participants agreed that this change is a positive development in the field. They acknowledged that “if you go back far enough, you can see why there’s very good reasons to have skepticism and concerns around research that was done” (12-R). They also noted that “you do not get changes in research trajectories quickly” (16-StSp), but felt that the field is “slowly improving” (07-AR), and that the current shift is “a change in the right direction” (05-StF).

#### Participatory research is “the way of the future”

As well as identifying a broad environment of change within the autism research field, participants identified a specific shift toward participatory research and increased involvement of autistic people in research: “I think it’s definitely shifting towards more involvement and more in every area, research priorities being set by autistic adults… being involved in the project from beginning to end rather than just as that participant” (17-RF). Participants believed that there exists “a growing body of researchers that do acknowledge the benefits and the value of participatory methods” (01-R), and felt that, despite some initial resistance (see subtheme 2.3) “some researchers might be realizing that they might be running out of a choice not to be inclusive” (12-R).

Participants attributed this shift toward participatory research to “a combination of [autistic people] pushing more and researchers finally realizing, ‘oh, maybe we should get their opinion on this’. I think that’s been making autistic individuals more involved in the research that’s supposedly being done for them” (05-StF). Another participant noted: “There’s just a lot more recognition of including the community in research, I think… autistic adults saying, ‘well, hang on a minute. You cannot do all this research without including us and asking us what we want’” (08-R).

Some participants had witnessed or contributed to increased community engagement within their own organizations, which is “very formalized now rather than being hap-hazard” (RSp-09); “my involvement in the project was part of that transformation of [the organization] moving from that tokenistic, ‘hey, look, we are training an autistic to be a researcher’, into that genuine respect and recognition and inclusion” (13-ARSp). Others noted a similar shift across the process of a research project, describing how “the participatory side of [the project], if anything, has continued to grow and increase and is actually a solid part of the project now” (01-R). Participants were confident that this evolution will continue, with one participant speaking optimistically about the future of community engagement in autism research:

I think that, going forward, every research project will have a participatory element. I think it is the way of the future and I think, in another 10 years’ time, to think that people used to do research projects to groups of people and those people were not involved, will just be a little bit absurd. Let us hope, anyway (01-R).

## Discussion

### How did stakeholders practice and experience participatory research within Autism CRC research projects?

In this study, we sought to understand the varied ways in which stakeholders practice and experience participatory research within projects supported by Australia’s Autism CRC. Consistent with previous research (Pellicano et al., 2014; Hollin and Pearce, 2019; Pickard et al., 2022), many of our academic participants spoke about the benefits of participatory research and believed that community members had made valuable contributions to their work. Like previous findings, and in line with evidence regarding participatory research beyond the autism field (Brett et al., 2014; Forsythe et al., 2019), community members’ insights were perceived to improve research outcomes and inform research findings that are more relevant to the autism community.

Yet, almost universally, participants’ experiences reflected community engagement consistent with a consultation approach, as opposed to the co-production approach endorsed by the Autism CRC. As with our own previous work (den Houting et al., 2021), community members were frequently described—both by academics and by community members themselves—as “advisors” who provided “feedback” to research teams, rather than as members of those research teams. In some cases, autistic people were employed as Research Assistants, which afforded community representation within research teams, albeit in subordinate roles. Academics largely retained decision-making power and control over the research process itself, and also over processes for community engagement, including decisions regarding how and when community members’ input was sought. Unsurprisingly, then, some community members felt that they had little or no influence over research processes and perceived their engagement as tokenistic. This finding echoes the sentiments of community members in Pellicano et al.’s study, who asked, “Whatever we say, is that really going to influence anyone?” (Pellicano et al., 2014, p. 4).

Consistent with previous research (Pellicano et al., 2014; Hollin and Pearce, 2019; Pickard et al., 2022), some of our participants faced challenges in communicating across different stakeholder groups, describing incidents of conflict and misunderstanding. In contrast to previous reports, though, our participants spoke of communication challenges occurring across a range of (autistic and non-autistic) stakeholder groups, and therefore tended not to attribute these difficulties to communication deficits on the part of autistic stakeholders. Instead, our participants perceived these communication challenges as arising from the varied experiences and perspectives that different stakeholders brought to the research process. In their Guidelines for the inclusion of autistic adults in research, Nicolaidis et al. (2019) warn against pathologizing autistic community members in instances of disagreement, noting that such challenges are usual in academic-community partnerships beyond the autism field. We echo this warning, and put forward our findings here as evidence that communication challenges in participatory autism research stem from factors far more complex and diverse than presumed autistic communicative “deficits.”

Discussion of the need for diversity in community representation is a theme consistently raised in previous relevant work (Pellicano et al., 2014; Hollin and Pearce, 2019; Pickard et al., 2022). In previous work, this emphasis on diversity perhaps stems from the perception that autistic people’s primary role in research is that of participant, with diversity and representativeness favorable within a participant group. When autistic people are instead engaged as research co-producers, however, there may be a tendency to erroneously apply these same expectations of diversity and representativeness. This is despite the striking lack of diversity evident among researchers—in our participant group, for example, a considerable majority of academic participants were white women. In our data, a focus on diversity was notably absent. Instead, many of our participants emphasized the importance of making research involvement accessible for community members, describing a range of different strategies that their research teams had implemented to facilitate the engagement of stakeholders with varying skills and expertise relevant to the particular research project. This finding signals an important distinction in how our participants approached the issue of diversity. Rather than implicitly placing the onus on the autistic and autism communities to make available a diverse range of representatives, this framing suggests that academics hold responsibility for providing a research environment that is accessible and welcoming to a range of collaborators (see Cascio et al., 2020 and Gowen et al., 2019, for discussion). The development of strong, trusting relationships between researchers and autistic community members should help to ensure that the accessibility needs of all team members are met.

Barriers to access, though, extend beyond the sensory processing differences, executive functioning difficulties, and other accessibility considerations discussed by our participants. In the autism field, the substance of research itself can serve as a considerable barrier to engagement by autistic people. Our participants spoke of autism research as being stigmatizing, irrelevant, alienating, and even harmful to autistic people. In writing about their experience as an autistic academic and activist, Botha (2021) describes regularly encountering dehumanizing, objectifying, and violent content and attitudes. Other autistic academics (Yergeau, 2013; Raymaker, 2019; Dwyer et al., 2021) have similarly described how the harmful and ableist nature of autism research has detrimentally impacted their experiences of academia, an experience that the first author of this manuscript shares. The autism research field is permeated by systemic violence against autistic people, ranging from ableist language (Bottema-Beutel et al., 2021) to efforts to prevent autism (e.g., Qiu et al., 2022). As oppressive as this is, autistic academics have the benefit of familiarity with the academic system, allowing us to engage with such content and attitudes from a position of relative privilege. For lay autistic people, engaging with such accounts may prove even more confronting. It is vital, therefore, that academics support their lay collaborators to safely engage with research and minimize exposure to offensive content, while at the same time working to enact systemic change to ensure that autism research can be safely accessed by all stakeholders.

### Why was participatory research practiced and experienced in these ways?

Within the academic setting, there exists an inherent power imbalance between researchers—who are typically highly educated, familiar with academic systems and structures, and perceived as “experts”—and lay community members, who may have few or none of these attributes. Also, community members in participatory research are often members of minority groups with little social power (e.g., Nicolaidis and Raymaker, 2015; McFarlane et al., 2022), an added complexity that may serve to exacerbate the power imbalance. One key aspect of participatory research is an epistemological shift—the ability to change the way we perceive community members and see them as experiential experts and equals, rather than as research participants or otherwise subordinate (Cargo and Mercer, 2008; Ocloo and Matthews, 2016).

Several of our themes and subthemes highlight the persistence of conventional perceptions regarding academics, academic systems, and the respective roles that researchers and community members can and should play within the research process; perceptions that are largely incompatible with meaningful community engagement. Participants across various stakeholder groups described the academic context, and academics themselves, as “out-of-touch” with the community. Participants described the priorities of the academic system as being disconnected from community priorities, and even as hindering efforts to ensure research has real-world applications. This finding mirrors closely the experiences of community members in Pellicano et al.’s (2014) study, who described research—and researchers—as “isolated” and “detached” (Pellicano et al., 2014, p. 9) from the practical issues faced by the autism community. This enduring perception of academia as an “ivory tower,” reported by both researchers and community members, is likely to reinforce existing power structures and serve as a barrier to meaningful community engagement in research.

Encouragingly, our participants often problematized the academic system, recognizing the limitations the “ivory tower” imposed. Even so, participants tended to accept current academic processes—including rigid protocols and tight constraints on time and funding—as inexorable and even necessary elements of the research process. Rather than highlighting the need for systemic change, participants spoke of adjusting participatory research processes to “fit” within the confines of academia, in some cases even suggesting that community members must also conform to these rigid academic standards if they are to contribute to knowledge production.

While participants expressed frustration with the academic orthodoxy, this orthodoxy was nonetheless evident in the roles that different stakeholders played within research processes. Some participants spoke candidly about the power imbalances that existed within their research teams and acknowledged these as inconsistent with best practices for participatory research. Lay community members’ roles were, at best, described as valuable but peripheral to the core research team; and, at worst, as disingenuous and tokenistic. Given this finding, it is evident that there remains a perception of community members—particularly autistic people, who have historically been limited to the role of participants in research—as less-than-equal contributors to research.

The establishment of strong working relationships between stakeholders is key in mitigating power imbalances. Current perceptions of community members may hinder the development of these relationships. Academics may be less motivated to build relationships with stakeholders who they perceive as being subordinate or ancillary to a research team, and reluctant to place trust in these stakeholders. Similarly, community members may have difficulty relating to and trusting “out-of-touch” academics, who they perceive as having limited understanding of the reality of lived experience. This may be particularly true for autistic community members, who have—as our participants noted—good reason to be wary of placing their trust in researchers. While these dynamics persist, participatory autism research teams will likely struggle to establish effective strategies for mitigating power imbalances. As a result, efforts to achieve genuine co-production of research may be hampered.

It is worth noting that several of our participants described research projects in which the only community members involved were autistic people employed as Research Assistants. Though participants typically perceived the employment of these Research Assistants as constituting community engagement, their descriptions suggested that these individuals’ roles might be more accurately framed as that of insider-researchers; that is, research staff (or students) who share an identity with the researched. Insider-researchers can bring considerable value to participatory research teams, occupying a “middle-ground” with access to both community and academic experiences, knowledge, and resources (Muhammad et al., 2015). There are, though, at least some aspects of a community member’s role that an insider-researcher will not typically be well-placed to undertake—for example, making judgements regarding accessibility for lay participants. Additionally, and crucially, a Research Assistant’s role ordinarily exists within a hierarchical academic team, with team members working under the supervision of a (most often non-autistic) Principal Investigator. Such a hierarchy inherently dictates the distribution of decision-making power and control within the academic team, with Research Assistants typically afforded relatively little of this power; equitable power sharing between the academy and the community is likely unachievable in these circumstances. It is vital, then, that participatory research teams do not rely solely on the expertise of insider-researchers, but include lay community members as equal partners in the research process.

### How might we improve participatory autism research going forward?

Consistent with previous research (Pickard et al., 2022), our participants described the autism research field as being in the midst of a broad shift in terms of attitudes toward autism and autistic people, with increasing recognition of autistic people as key stakeholders in the production of knowledge about autism. Although they often found participatory research challenging in practice, participants invariably expressed positive attitudes regarding community engagement in autism research. This context of attitudinal change toward autistic people and widespread support for community engagement provides a rich opportunity to advance participatory autism research.

To move toward a more participatory future in autism research, it is important also to recognize the historical and ongoing epistemic injustice practiced against autistic people. Broadly, epistemic injustice refers to a range of injustices carried out against a person in their capacity as a knower or a producer of knowledge; see Catala et al. (2021) for a detailed discussion of the many types of epistemic injustice autistic people face. In short, autistic people face both testimonial injustice, in which biases against autistic people serve to diminish their credibility as epistemic agents; and hermeneutical injustice, in which the epistemic resources (e.g., concepts and language) necessary for autistic people to understand and articulate their experiences are lacking (Fricker, 2007; Catala et al., 2021; Dinishak, 2021). Addressing this epistemic injustice is both a necessary precondition for, and a likely result of, effective participatory autism research. Autistic people must be recognized as credible producers of knowledge if they are to contribute to participatory research; that is, testimonial injustice must be addressed. At the same time, autism research produced by and/or with autistic people will likely be better placed to produce the epistemic resources needed to overcome hermeneutical injustice. This is evidenced by, for example, Milton’s Double Empathy Problem (Milton, 2012), and recent co-produced and autistic-led work on autistic burnout (Raymaker et al., 2020; Higgins et al., 2021) and autistic inertia (Buckle et al., 2021; Phung et al., 2021), all of which have introduced to the academic literature new epistemic resources for understanding autistic experiences.

As discussed above, our participants predominantly adopted a consultative approach to community engagement in research. While this approach lacks the power-sharing that is central to more genuinely participatory work, it can nonetheless serve as a key foundational step in establishing an equitable co-production partnership. Consultation between academics and community members can provide a valuable opportunity to establish working relationships and identify shared values and interests. To move beyond consultation, it will be critical to build upon these incipient relationships, working over time to establish effective communication and mutual trust.

As our participants explained, effective participatory research must be “set up for success,” with community engagement intentionally established as core to the research from the earliest stages of a project. We suggest that, to be most beneficial, community engagement can be established even earlier. Rather than approaching community engagement as subsumed within a particular research project, we encourage researchers to consider community engagement as an independent—but equally important—process. Ideally, stakeholders could first work to establish trusting relationships, open communication, and processes for power-sharing, before collaboratively identifying research topics of mutual interest and beginning the process of research co-production. The Academic Autism Spectrum Partnership in Research and Education (AASPIRE) is an example of a long-term participatory partnership that has successfully followed this approach (Nicolaidis et al., 2011, 2019).

Establishing and maintaining participatory partnerships of this type can require considerable resourcing. As our participants observed, existing academic systems are often designed to facilitate conventional research processes, and lack the flexibility to accommodate participatory research. To foster meaningful community engagement in research, then, change is needed at the systemic level. Our participants noted three elements of current academic systems that serve as barriers to participatory research: academic protocols and rules; time pressures; and funding constraints. We suggest that two overarching changes within academic systems are necessary to mitigate these barriers. First, greater flexibility and responsivity within the academy, allowing for the tailoring of protocols, timelines, and budgets to better accommodate a range of research processes and stakeholders. Second, cultural change, promoting greater recognition of the value of community engagement in research, and ensuring that the additional labor inherent in such work is acknowledged and rewarded.

### Limitations

While this study provides valuable insights into stakeholders’ experiences of community engagement in Australian autism research, these findings must be considered in the context of the study’s limitations. First, participation in this study was open to any stakeholder involved in producing Autism CRC-funded research, regardless of their level of experience with participatory research. It is therefore possible that our participants were motivated to take part due to a particular interest in the topic of participatory research, which may be reflected in our findings. As a result, the extent and nature of the challenges identified herein may well be an *underestimate* of those experienced in autism research more broadly.

Second, although we did not specifically recruit participants for their participatory research experience, we asked each participant to identify and describe a participatory autism research project in which they had been involved. While every participant was able to identify and discuss a project they perceived as participatory, some participants noted that they felt their projects were not “good” examples of participatory research, and others described projects with minimal or no community engagement in the research process. Consistent with our previous work (den Houting et al., 2021), participants in this study had varied understandings of what constitutes participatory research, which did not always align with accepted definitions. As a result, the findings presented here describe participants’ experiences of research that they perceived as participatory, some of which appeared to lack meaningful community engagement.

Third, our recruitment process relied on Autism CRC Project Leaders to nominate members of their project team/s as potential participants. To encourage the nomination of a range of different stakeholders, we specified that project teams may comprise both academic and community members, in paid or unpaid roles, including advisors and consultants. Despite such encouragement, most nominated participants were academics (see den Houting et al., 2021). Only two of the current participants held exclusively non-academic roles in the autism community (02-A, an autistic person; and 10-F, a family member/career); the remaining 18 participants all held academic roles, either exclusively or in addition to non-academic roles. As a result, our findings provide only limited insight into lay community members’ experiences of participatory autism research. In addition, most participants were women, white, highly educated, and engaged in full-time employment or study. Given that previous findings indicate considerable disparity between academic and community experiences of autism research (Pellicano et al., 2014), future studies should make additional efforts to ensure that the perspectives of non-academic community members, particularly those from marginalized communities, are represented.

## Conclusion

The findings presented here paint a picture of a field in flux, facing a shift from the “normal science” (Pellicano and den Houting, 2022) of the ivory tower to a more inclusive, real-world paradigm with community members valued as key agents in knowledge production. It is clear, though, that much remains to be done if the field of autism research is to achieve epistemic justice for all stakeholders. At an individual level, we must continue working to forge meaningful, sustainable academic–community partnerships to facilitate the power-sharing that is key to genuine research co-production. At a systemic level, considerable change is needed to eliminate the barriers that hinder community engagement in research. By moving forward with such changes, we may indeed find that participatory research is “the way of the future.”

## Data availability statement

The datasets presented in this article are not readily available because the data reported in this study are qualitative interview data. The raw data include details that could identify participants. Participants did not consent to sharing of identifiable data beyond the current research team. Requests to access the datasets should be directed to jac.denhouting@mq.edu.au.

## Ethics statement

This study received ethical approval from Macquarie University Human Research Ethics Committee (Project ID 3300). Written informed consent was obtained from all participants for their participation in this study.

## Author contributions

JH, KI, and JM contributed to the conception and design of the study. EP contributed to the conception, design, and data analysis. JdH contributed to the conception and design, conducted all interviews, led data analysis, and wrote the first draft of the manuscript. All authors contributed to the article and approved the submitted version.

## Funding

The authors acknowledge the financial support of the Cooperative Research Centre for Living with Autism (Autism CRC), established and supported under the Australian Government’s Cooperative Research Centers Program. This work was also supported by a Macquarie University Research Fellowship awarded to JdH.

## Conflict of interest

The authors declare that the research was conducted in the absence of any commercial or financial relationships that could be construed as a potential conflict of interest.

## Publisher’s note

All claims expressed in this article are solely those of the authors and do not necessarily represent those of their affiliated organizations, or those of the publisher, the editors and the reviewers. Any product that may be evaluated in this article, or claim that may be made by its manufacturer, is not guaranteed or endorsed by the publisher.
