# Supplementary material for: From ivory tower to inclusion: Stakeholders’ experiences of community engagement in Australian autism research
Source: Front Psychol. 2022 Aug 25;13:876990. doi: 10.3389/fpsyg.2022.876990 (PMC9454607; doi:10.3389/fpsyg.2022.876990)
Supplement: Supplementary file 1 [file Data_Sheet_1.DOCX]

**Supplementary Material - Interview template**

**Introduction and consent check**

Thank you for agreeing to chat to me today.

The research that I’m working on is looking at participatory research in the Autism CRC. I will be interviewing academic and community partners about their opinions and experiences of participatory autism research.

I’m going to start by asking you some questions about yourself. Then I’ll ask about your thoughts and experiences of participatory autism research in relation to a specific research project, and also more generally. You can think about the same research project you were thinking about when you completed the survey, or a different project, as long as it’s an Autism CRC project. There are no right or wrong answers to any of these questions, I’m just interested in your experiences.

All the answers you give me will be kept private, and I won’t use your real name when I write or talk about the interviews later on. If at any point you want to stop, or skip a question, just let me know.

If it’s okay with you, I will record today’s interview. The recording will be sent to a company to be transcribed. The only people who will hear the recording will be the research team and the transcriber. Is that okay?

*If yes* - Proceed

*If no* – Are you happy to talk to me without being recorded?

*If yes –* Proceed without recording

*If no* – Stop interview – consent denied.

**Demographics**

**Tell me a bit about yourself**

Probe questions:

1. What is your current role?
2. What experience do you have in autism research?
3. What other experience do you have with autism professionally/personally?

**Attitudes**

**What does the phrase “participatory research” mean to you?**

Probe questions:

1. What are some examples of research processes that you would consider participatory? For example, is disseminating results from research to autistic people participatory? What about consulting with the autism community? Or engaging with the autism community as partners in the research process?
2. What do you think are the key elements that separate participatory research from non-participatory research?

**Research experience**

**I’m going to ask you to think about a specific Autism CRC research project that you have been involved in, that in your view was participatory research. It can be the same project that you thought about when you completed the online survey, or a different project. It could be a good experience, a bad experience, or somewhere in between. Tell me about your experience of the research project.**

Probe questions:

1. What was your role in the research project?
2. In what way were community partners involved in the research project?
   1. In what stages of the project were they/you involved? What did they/you do?
   2. For academic partners: How did you ensure that community partners were involved? Do you think they felt involved in the research?
   3. For community partners: How did academic partners ensure that you/community partners were involved in the research? Did you feel genuinely involved? In what way?
3. Which factors were most important in shaping your experience of the research project?

*If the participant only identifies a very negative or very positive example:*

1. Have you had any good/bad experiences of participatory research? Tell me about those.

**Relationships**

**In other research, people have had varying experiences of the relationships between community and academic partners. Tell me about the relationships between different academic and community partners in your specific research project.**

Probe questions:

1. Did you feel like some partners were valued more than others? What did this look like?
2. Were any steps taken to counteract this imbalance? For example, accommodations for autistic partners?
3. Did different partners seem open to learning from each other? In what ways did different partners learn from each other during the research process?
4. How did your project team manage conflict between partners during the research process?

**Outcomes**

**What were the outcomes of your research project, academically and personally?**

Probe questions:

1. How do you think community engagement impacted on the outcomes of the project? How do you know this? How do you think we might measure this impact?
2. What were the challenges of engaging community partners in this project?
3. Are there any steps that could be taken in the future to improve the meaningful engagement of community partners in autism research?
4. What were the benefits of having community partners engaged in this project?

**Systemic context**

**Thinking about autism research generally now, what are the factors that you think influence how autism research gets done?**

Probe questions:

1. How do you think researchers traditionally perceive autistic peoples’ roles in research?
2. How do you think autistic people traditionally perceive autism research?
3. Do you think community partners are ready and able to work with academic partners? Do you think academic partners are ready and able to work with community partners?
4. What would it take to make participatory research easier for you? Do you think you can access what you need?
5. Would you be interested in a training program on participatory research? What might this look like?

That’s all of my questions. Is there anything else you would like to add? Do you have any questions for me? Thank you very much for sharing your thoughts and experiences with me.

What will happen next is that I will send the recording of our interview to be transcribed, and I will send you a copy of the transcript so that you can check it is accurate. Liz and I will analyse all of the transcripts from all the interviews, and then we will write a paper describing the results. If we want to include any of your quotes in the paper, we will check with you first. If you are interested, we can send you a copy of any papers we write based on these interviews.

Feel free to contact me via email (jac.denhouting@mq.edu.au) or phone if you think of any other questions or comments. Thanks again.
